# Establishment of a humanized mouse model of pulmonary fibrosis for advancing drug validation strategies

**DOI:** 10.1038/s41598-025-18370-7

**Published:** 2025-10-06

**Authors:** Md. Nakibul Hasan, Yuri Chae, Su Yeon Lee, Young Mo Kang

**Affiliations:** 1 Division of Rheumatology, Department of Internal Medicine, Kyungpook National University School of Medicine, Daegu, South Korea; 2 Preclina Inc., 719 & 1302, Teratower B, 167, Songpa-daero, Songpa-gu, Seoul, South Korea

**Keywords:** Idiopathic pulmonary fibrosis, Humanized mice, Bleomycin-induced lung fibrosis, Nintedanib, Immunology, Diseases

## Abstract

Idiopathic pulmonary fibrosis (IPF) is a progressive interstitial lung disease characterized by excessive extracellular matrix deposition and limited therapeutic options. Conventional preclinical models, such as the bleomycin (BLM)-induced lung fibrosis (BILF) model in immunocompetent mice, fail to replicate the complexity of the human immune environment and are thus unsuitable for evaluating human-specific antibody and cell therapies. To overcome this limitation, we developed two humanized mouse models with functional human immune components: one engrafted with peripheral blood mononuclear cells (PBMCs) and the other with hematopoietic stem cells (HSCs). Pulmonary fibrosis was induced using BLM in both humanized models. In the PBMC-humanized model, human T cells accounted for 79.9% ± 9.7% of CD45^+^ cells; however, this model was limited by high incidence of graft-versus-host disease (GvHD). In contrast, the HSC-humanized model demonstrated stable multilineage engraftment (T cells: 20.1% ± 28.3%; B cells: 56.8% ± 25.4%), absence of GvHD, and consistent fibrotic pathology. Both models exhibited increased inflammatory infiltration, fibroblast activation, and collagen deposition following BLM induction. Nintedanib treatment significantly ameliorated fibrotic changes in both models. These findings establish the HSC-humanized BILF model as a clinically relevant platform for investigating human immune responses in IPF and for preclinical evaluation of human-targeted therapies.

**Supplementary Information:**

The online version contains supplementary material available at 10.1038/s41598-025-18370-7.

## Introduction

Idiopathic pulmonary fibrosis (IPF) is a chronic and progressive lung disease with unknown etiology that is characterized by the histopathological pattern of usual interstitial pneumonia (UIP). The median survival duration following IPF diagnosis ranges from 2 to 5 years^1^. UIP is characterized by epithelial cell proliferation, basement membrane degradation, and fibroblastic foci^2^. Animal models that have been used to study the pathogenesis of IPF and other fibrotic lung diseases do not exhibit progressive disease and often cannot replicate the histological features of UIP or other interstitial lung disorders. However, they remain crucial in PF research, providing valuable insights into lung injury, inflammation, and fibroproliferation^3^.

Various conventional and recently developed experimental models, such as those developed through radiation-induced damage, bleomycin (BLM) administration, and transgenic animals or gene transfer using fibrogenic cytokines, have generated important insights into the underlying disease mechanisms and helped identify novel therapeutic targets for evaluation in clinical trials^4–6^. Of these models, the BILF model is the most commonly used for investigating fibrosis etiology and progression because of its simplicity, reproducibility, and affordability. However, it cannot replicate key human disease features, such as the chronic and progressive nature of UIP^7^.

Although traditional animal models have been invaluable, the applicability of findings in clinical practice is constrained by species-specific differences in immune responses and molecular pathways^8,9^. For example, antibody therapeutics that are engineered to target epitopes unique to human antigens lack compatibility with animal antigens due to their high specificity. Furthermore, the humanization of antibodies limits their suitability for interaction with conventional animal models, making the assessment of their clinical efficacy difficult^10,11^. Regarding cell therapies, structural differences in immunosuppressive molecules between humans and mice cause incompatibilities in the binding between human and mouse cells^12^. Therefore, cross-reactivity between human mesenchymal stem cells or other cell therapies and animal target cells in conventional animal models is unknown, which makes the accurate representation of the physical interactions between human mesenchymal stem cells and immune cells difficult^13^. However, the evolution of medical and pharmacological technologies is facilitating the development of complex, highly targeted, and personalized treatments. These therapies often require validation within an environment containing essential elements of the human biological system. Given the differences between mouse and human biology as well as the human-specific nature of particular drugs and diseases, choosing animal models that closely mimic the complexities of the human biological system is crucial for disease modeling and preclinical assessment^14^. Furthermore, despite the differences between mice and humans and the technical constraints of in vivo studies of human biology, the demand for the use of animal models in studying human cells, tissues, and organs without endangering individuals is increasing^15,16^.

Humanized mouse models were developed to overcome these limitations and offer a more representative system for studying human disease mechanisms^17^. These models are established by engrafting immunodeficient mice with human cells or tissues, enabling the study of human-specific immune responses^18^. Several models of humanized PF have been established; these models commonly incorporate either lung fibroblast cells or peripheral blood mononuclear cells (PBMCs) derived from patients with lung fibrosis for the induction of fibrosis in immunodeficient mice^19,20^. These models offer insights into the roles of different groups of fibroblasts and demonstrate the role of epithelial–fibroblast interactions in the absence of immune cells. However, these models may not fully represent human lung fibrosis in which immune cells play a significant role^7^. A BLM-induced PF model was successfully established using PBMC-reconstituted humanized mice^13^. However, graft-versus-host disease (GvHD) developed approximately 5 weeks after PBMC reconstitution, with spontaneous lung fibrosis developing by 8 weeks in mice^21,22^. These features highlight the need for alternative models that can circumvent this issue, such as those that use hematopoietic stem cell (HSC)-humanized mice.

To date, no definitive treatments have been developed for PF, with lung transplantation being the only curative clinical option^23^. The introduction of antifibrotic agents, such as nintedanib and pirfenidone, has shifted the landscape of IPF therapeutic management. These agents inhibit disease progression by targeting the key signaling pathways involved in fibrogenesis^24,25^.

The present study successfully established and validated a robust PF model using humanized mice, offering a novel testing platform for human target-specific antibody therapeutics while eliminating the need for primate models. We employed two humanization approaches: PBMC-humanized mice, which had a restricted study window of 5 weeks to mitigate GvHD, and an HSC-based model (HSC-humanized mice), which demonstrated the multilineage development of human immune cells. These humanized models were used to establish a BILF system that mimicked the pathophysiological and immune responses of human lung fibrosis. The therapeutic efficacy of nintedanib, a human antifibrotic drug, was evaluated using these models to validate our BILF humanized mouse model. To the best of our knowledge, this study is the first to develop a BILF model using HSC-humanized mice and multilineage stem cells. This model provides a robust platform for studying PF and evaluating antifibrotic therapies.

## Methods

### Mice

The experimental procedures and animal care protocols were conducted in line with the institutional animal welfare protocol and with the approval of the Kyungpook National University Institutional Animal Care and Use Committee. The findings of this study were reported in accordance with the ARRIVE guidelines for experimental reporting^26,27^. Adult male and female NOD.Cg-*Prkdc*^*scid*^*IL2γg*^*tm1 Sug*^/JicKoat (NOG) mice were purchased from Koatech, South Korea. The animals were fed commercial food pellets and provided with acidic water (pH 2.5–3.0) ad libitum to inhibit infection by *Pseudomonas* spp.

### PBMC isolation from the donor blood sample

PBMCs were isolated from blood samples collected from healthy donors who provided informed consent. Blood was collected in EDTA-coated tubes (BD Microtainer), diluted 1:1 with Dulbecco’s phosphate-buffered saline, and layered over Ficoll-Paque™ PLUS density gradient media (3:5; GE Healthcare). Following centrifugation at 400×*g* for 30 min at room temperature (breakoff), the buffy coat containing PBMCs was collected. The cells were washed with Roswell Park Memorial Institute (RPMI) 1640 medium (Lonza), centrifuged at 400×*g* for 5 min, and resuspended in cryopreservation medium before storage in liquid nitrogen.

### Isolation of CD34^+^ cells from umbilical cord blood (UCB)

UCB samples were collected and centrifuged at 400×*g* for 10 min at room temperature. The supernatant was discarded. The blood pellet was resuspended in phosphate-buffered saline, layered onto Ficoll-Paque™ PLUS density gradient media (3:5), and centrifuged at 400×*g* for 30 min at room temperature. The buffy coat containing mononuclear cells was collected, washed with RPMI medium, and counted using a Countess 3 cell counter (Invitrogen). Up to 1 × 10^8^ cells were blocked with human Fc receptor blocking buffer, labeled with CD34 MicroBeads, and separated magnetically using the MACS system (Miltenyi Biotec, Germany). Purified CD34^+^ cells were washed, counted, and preserved in freezing medium until use.

### Generation of humanized mice

We used PBMCs from healthy donors and HSCs from UCB to generate humanized mice.

**PBMC-humanized mice:** For PBMC humanization (PreHu-PC mice), 6-week-old, male NOD.Cg-*Prkdc*^*scid*^*IL2γg*^*tm1 Sug*^/JicKoat (NOG) mice were administered with 1 × 10⁷ PBMCs, which were resuspended in 100 µL RPMI medium without irradiation, via tail vein injection.

**HSC-humanized mice:** For HSC humanization (PreHu-HER), we used 4-day-old irradiated pups for intrahepatic injection following previously described procedures with slight modifications^28,29^. In brief, on the day of injection, thawed HSCs were resuspended in 50 µL phosphate-buffered saline and administered via intrahepatic injection at a dose of 3 × 10^4^ CD34^+^ cells per mouse.

### Establishment of a BLM-induced lung fibrosis model using humanized mice

Following the successful generation of humanized mice, which was confirmed through blood fluorescence-activated cell sorter (FACS) analysis, a fibrosis model was established by administering BLM. Oropharyngeal aspiration (OA) was performed to administer BLM as described previously with slight modification^30^. In brief, mice were anesthetized using isoflurane (Hana Pharm) inhalation and positioned on a curved board. Then, the BLM solution was administered onto the back of the tongue. The experimental groups were as follows: Normal ⇒ Humanized Control (PBMC/HSC-humanized mice without bleomycin administration), Vehicle ⇒ Humanized BILF Control (PBMC/HSC-humanized mice with bleomycin administration and treated with vehicle), and nintedanib ⇒ Humanized BILF nintedanib (PBMC/HSC-humanized mice with bleomycin administration and treated with 40 mg/kg nintedanib from day 7 to 21, once daily by oral gavage).

### FACS analysis

The single-cell suspension of the mouse peripheral blood and BALF were harvested, and 1 × 10^6^ cells were used for FACS staining. The cells were then washed with washing buffer composed of 0.2% bovine serum albumin in phosphate-buffered saline and centrifuged at 5000 rpm at 4 °C for 5 min. The supernatant was discarded, and 10 µL of human Fc receptor blocking buffer was added to the cells, which were then incubated with antibodies for 30 min at 4 °C. The antibodies used in this study are listed in Supplementary Table 1. Following incubation with antibodies, the cells were washed and subsequently fixed with 100 µL of 2% paraformaldehyde (PFA). Finally, the stained and fixed cell suspension was placed into a 96-well plate for analysis using a NovoCyte™ Flow Cytometer (Agilent Technologies).

### Sampling of the bronchoalveolar lavage fluid (BALF) and lung tissue

At 21 days after BLM administration, the mice were euthanized in a CO_2_ gas chamber. After collecting the blood, the mice were pinned to the surgical board. The BALF was collected as described previously with some modifications^31^. In brief, the skin was incised to reveal the salivary glands, which were isolated using pincers to reveal the sternohyoid muscle. The muscle surrounding the trachea was dissected with forceps to visualize the trachea. The middle of the exposed trachea was pierced precisely within two cartilage rings using a 20-G needle. A catheter was inserted into the trachea. The solution was gently aspirated three times, with 1 mL each time, and maintained on ice until subsequent analysis.

The left lung was harvested for micro-computed tomography (CT) imaging and subsequent histopathological analysis, whereas the right lung was snap-frozen in liquid nitrogen for hydroxyproline assay.

### Micro-CT analysis

Lung specimens were prepared for micro-CT imaging as described previously^32^. In brief, the lung specimens were fixed in 4% PFA and then incubated overnight in 15% sucrose solution. On the next day, the lung specimens were incubated in 70%, 80%, and 90% ethanol for 2 h each and then in 100% ethanol overnight. Subsequently, the lung specimens were used for micro-CT. The three-dimensional (3D) images of the lung specimens were analyzed to quantify the fibrosis volume and percentage using a 3D slicer.

### Histopathology and immunohistochemistry analysis

After micro-CT, the lung tissues were processed and subsequently embedded in paraffin and sliced into 3-µm sections for hematoxylin and eosin (H&E) and Masson’s trichrome (MT) staining. The modified Ashcroft’s and fibrosis scores were calculated as described previously and listed in Supplementary Tables 2 and 3^33,34^. The modified Ashcroft’s score was assessed using H&E staining images, whereas the fibrosis score was determined using MT staining images, with four fields from each section used for both assessments. Two experienced researchers calculated both scores blindly.

Paraffin-embedded lung and spleen sections were used for immunohistochemical (IHC) staining. In brief, the sections were deparaffinized for 1 h at 60 °C, dewaxed in xylene, and rehydrated using an ordered sequence of ethanol solutions. Antigen retrieval via heat was performed using a TintoRetriever Pressure Cooker (BioSB) with Tris–EDTA buffer (10 mM Tris base, pH 9.0) for 10 min at low pressure. Following a 30-min cooling period at room temperature, the sections were blocked using 5% normal swine serum for 20 min, followed by 1-h incubation with the primary antibody (Supplementary Table 4) at room temperature. After incubation of the sections with secondary antibody and peroxidase blocking, the signal was amplified and visualized using a 3,3′-diaminobenzidine substrate and chromogen system (Dako, Agilent Technologies Inc., USA). All stained slides were scanned using the Motic Easy Scanner (Motic Asia, Hong Kong), and the figures obtained were exported for subsequent scoring and quantification.

### Statistical analysis

All data are expressed as mean ± standard error of the mean or mean ± standard deviation, as specified. Differences between groups were analyzed using Student’s t-test for independent samples or one-way analysis of variance with Tukey’s post hoc test for multiple comparisons. Statistical analyses were performed using IBM SPSS Statistics software (version 26.0; IBM Corp, Chicago, IL, USA). P-values were two-tailed, with statistical significance denoted as follows: *P < 0.05, **P < 0.01, and ***P < 0.001. Graphs and visual representations of the data were created using GraphPad Prism.

## Results

### Validation and characterization of PBMC-humanized mice

**Fig. 1:**
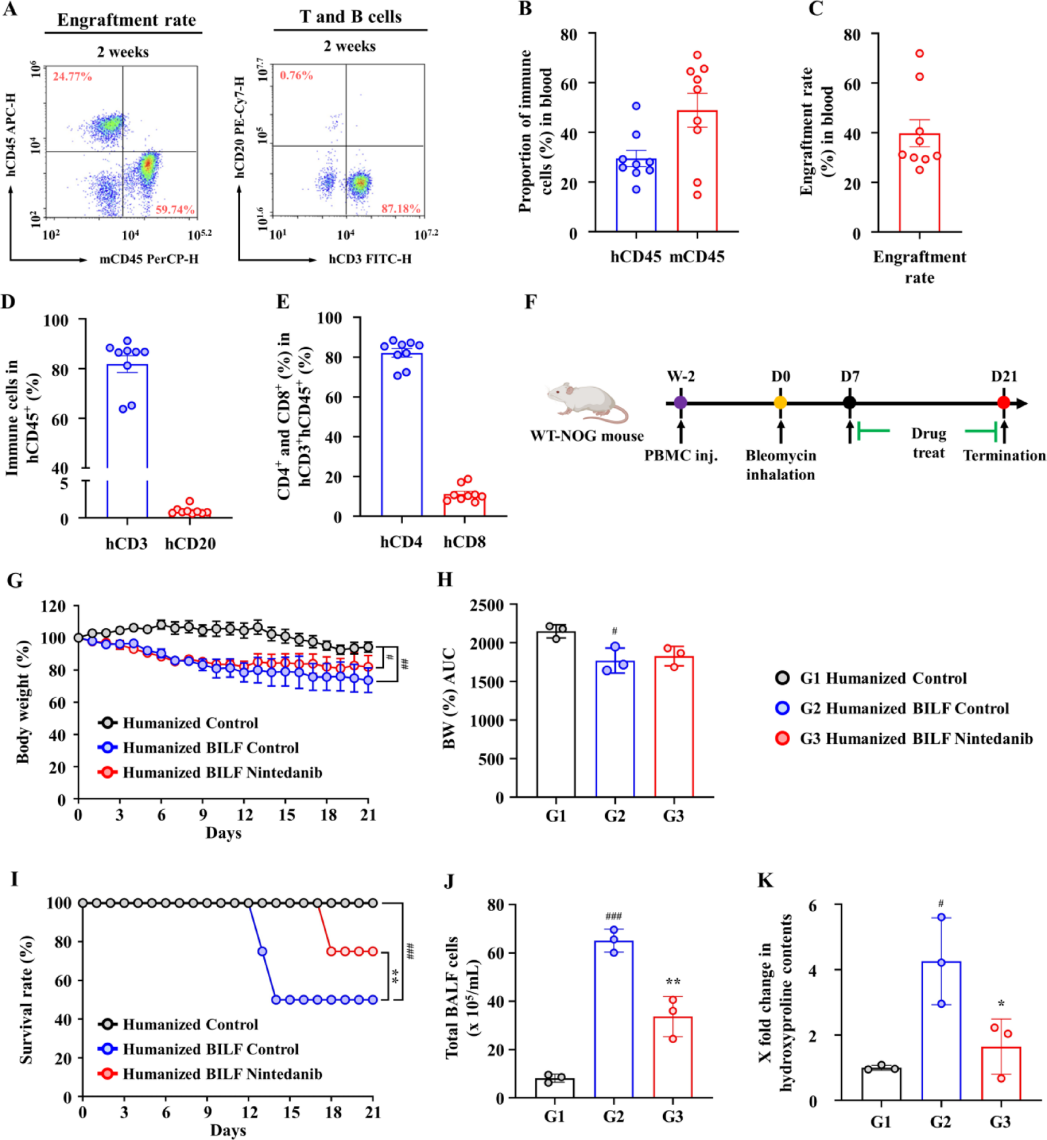
Evaluation of immune cell humanization and generation of the BILF model in PBMC-humanized mice. (**A**) Representative flow cytometry gating strategy for peripheral blood at 2 weeks after PBMC injection. (**B**) Proportions of human CD45^+^ cells and mouse CD45^+^ cells in blood. (**C**) Engraftment rate efficiency (% Graft) was calculated as the proportion of human CD45^+^ cells in the total CD45^+^ cell population (human and mouse) in peripheral blood. (**D**) Flow cytometry analysis confirming the presence of human leukocyte subsets, including T cells (hCD3^+^) and B cells (hCD20^+^) within the human CD45^+^ cell fraction, with T cells constituting the dominant population. (**E**) Distribution of human CD4^+^ and CD8^+^ T cell subsets within the hCD3^+^ cell population. (**F**) Schematic illustration of the experimental timeline, including the humanization process, bleomycin administration, and subsequent nintedanib treatment. (**G**) Body weight changes (%) relative to day 0 in the normal-, vehicle-, and nintedanib-treated groups over time. (**H**) Area under the curve (AUC) analysis of body weight percentage (BW%) changes. (**I**) Survival analysis comparing the vehicle and nintedanib treatment groups after bleomycin administration. (**J**) Total cell count data in the BALF. (**K**) Quantitative expression level (fold change compared with the normal group) of the hydroxyproline content in BILF lung tissue. Data are expressed as mean ± standard error of the mean. *P < 0.05, **P < 0.01, and ***P < 0.001 versus vehicle; ^#^versus the normal group. BILF, bleomycin-induced lung fibrosis; PBMCs, peripheral blood mononuclear cells; BALF, bronchoalveolar lavage fluid.

Prior to BLM administration, the humanization level was evaluated using FACS analysis 2 weeks after PBMC injection. Six-week-old male NOG mice were intravenously injected with PBMCs, and the analysis confirmed the successful engraftment of hCD45^+^ human leukocytes (Fig. 1A,B), with an engraftment rate of (30.42% ± 18.12%) (Fig. 1C). T cells constituted the majority of the engrafted cells (79.99% ± 9.72%) (Fig. 1D), with the overall engraftment rate exceeding 25%. As shown in (Fig. 1E), the hCD3^+^ T cell population was further characterized into CD4^+^ and CD8^+^ subsets.

These results were consistent with those of a previous study^35^, validating that the mice were successfully humanized prior to BLM induction.

### Establishment of a PF model using PBMC-humanized mice

To replicate human PF, a fibrosis model was established by administering BLM to humanized mice stably reconstituted with human PBMCs. The timeline of the humanization and treatment process is illustrated in (Fig. 1F). Following BLM administration, the body weight percentage (Fig. 1G) and area under the curve of the body weight percentage (Fig. 1H) of humanized mice decreased progressively, with significantly greater weight loss than that observed in the untreated control group. The BLM-treated group exhibited a pronounced decrease in survival rate (%) when assessed by survival analysis, relative to controls (Fig. 1I). BALF analysis revealed a significant increase in total cell counts in the BLM-treated group (65.10 ± 4.77) compared with the normal group (8.13 ± 1.67) (Fig. 1J). Hydroxyproline levels in the BLM-treated group were elevated by (4.26 ± 0.77)-fold compared with those in the control group, indicating enhanced collagen production (Fig. 1K).

**Fig. 2:**
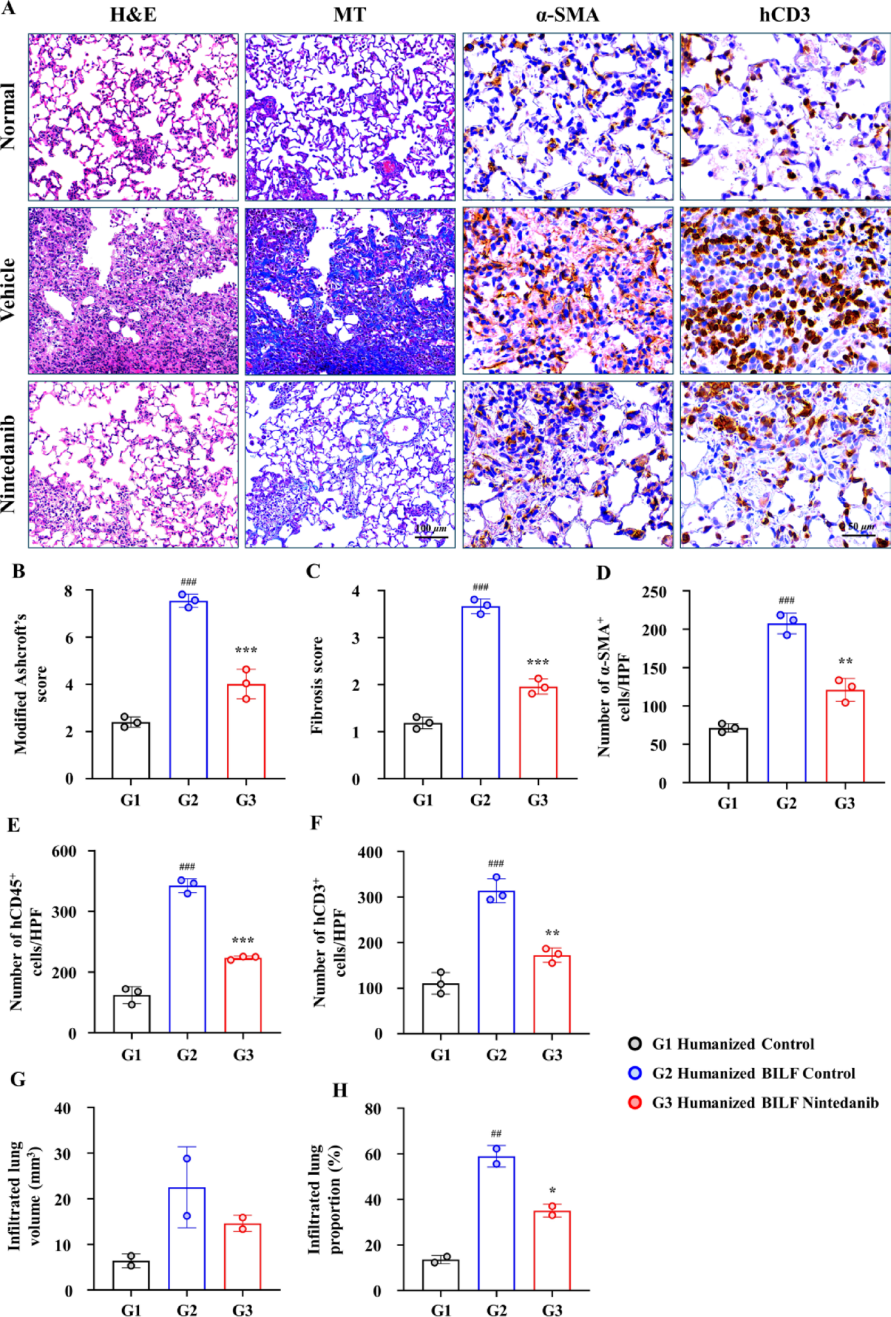
Histopathological and micro-CT analysis of lung tissue from PBMC-humanized mice 21 days after BILF induction. (**A**) Representative hematoxylin and eosin (H&E) and Masson’s trichrome (MT) staining for lung tissues as well as immunohistochemical (IHC) staining of α-SMA and hCD3 at magnification 200 × for H&E and MT and 400 × for IHC. Scale bars: 100 µm (H&E and MT) and 50 µm (IHC). (**B** and **C**) Quantification of lung fibrosis severity: the modified Ashcroft’s score (H&E stain) and fibrosis score (MT stain) showing the extent of fibrotic changes. (**D**) Quantification data of α-SMA^+^cells/high-power field (HPF). (**E** and **F**) Quantification of human immune cells, including hCD45^+^ leukocytes and hCD3^+^ T cells, expressed as the number of cells/HPF. (**G**) Quantification of the infiltrated lung volume, indicative of fibrosis volume (mm^3^). (**H**) Infiltrated lung proportion calculated as the ratio of infiltrated lung volume to total lung volume. Data are expressed as mean ± standard error of the mean. *P < 0.05, **P < 0.01, and ***P < 0.001 versus vehicle; ^#^versus the normal. BILF, bleomycin-induced lung fibrosis; PBMCs, peripheral blood mononuclear cells; BALF, bronchoalveolar lavage fluid.

Histological examination of lung tissues revealed significantly higher Ashcroft’s scores (7.64 ± 0.09) and fibrosis scores (3.67 ± 0.09) in the BLM-treated group than in the control group, indicating severe PF (Fig. 2A–C). IHC analysis revealed substantial infiltration by α-SMA^+^ cells (207.58 ± 7.84) in the lung tissue of the BLM-treated group, indicating active fibrogenesis and myofibroblast activation (Fig. 2D). Moreover, IHC analysis for human-specific markers (hCD45 and hCD3) confirmed the presence of human immune cell infiltration within the lung tissue (Fig. 2E–F). Furthermore, ex vivo micro-CT analysis revealed extensive infiltration of fibrotic changes in the lungs (Fig. 2G,H, Supplementary Fig. 1A–B).

Notably, the Normal (humanized control) group also exhibited features of GvHD and spontaneous baseline fibrosis. These included a Modified Ashcroft’s score (2.40 ± 0.13) (Fig. 2B), fibrosis score (1.19 ± 0.07) (Fig. 2C), and infiltration by human immune cells, with hCD45^+^ cells (124.33 ± 15.99 per HPF) and hCD3^+^ cells (110.58 ± 13.60 per HPF) (Fig. 2E,F). Micro-CT analysis further confirmed the presence of measurable fibrotic tissue (13.60 ± 1.80%) in the Normal group (Fig. 2H).

Collectively, these results confirm that BLM successfully induced PF in the PBMC-humanized mouse model, thereby effectively mimicking the pathophysiological progression of lung fibrosis observed in humans, while also revealing that humanized control mice develop GvHD-associated baseline fibrosis.

### Generation and characterization of HSC-humanized mice

**Fig. 3:**
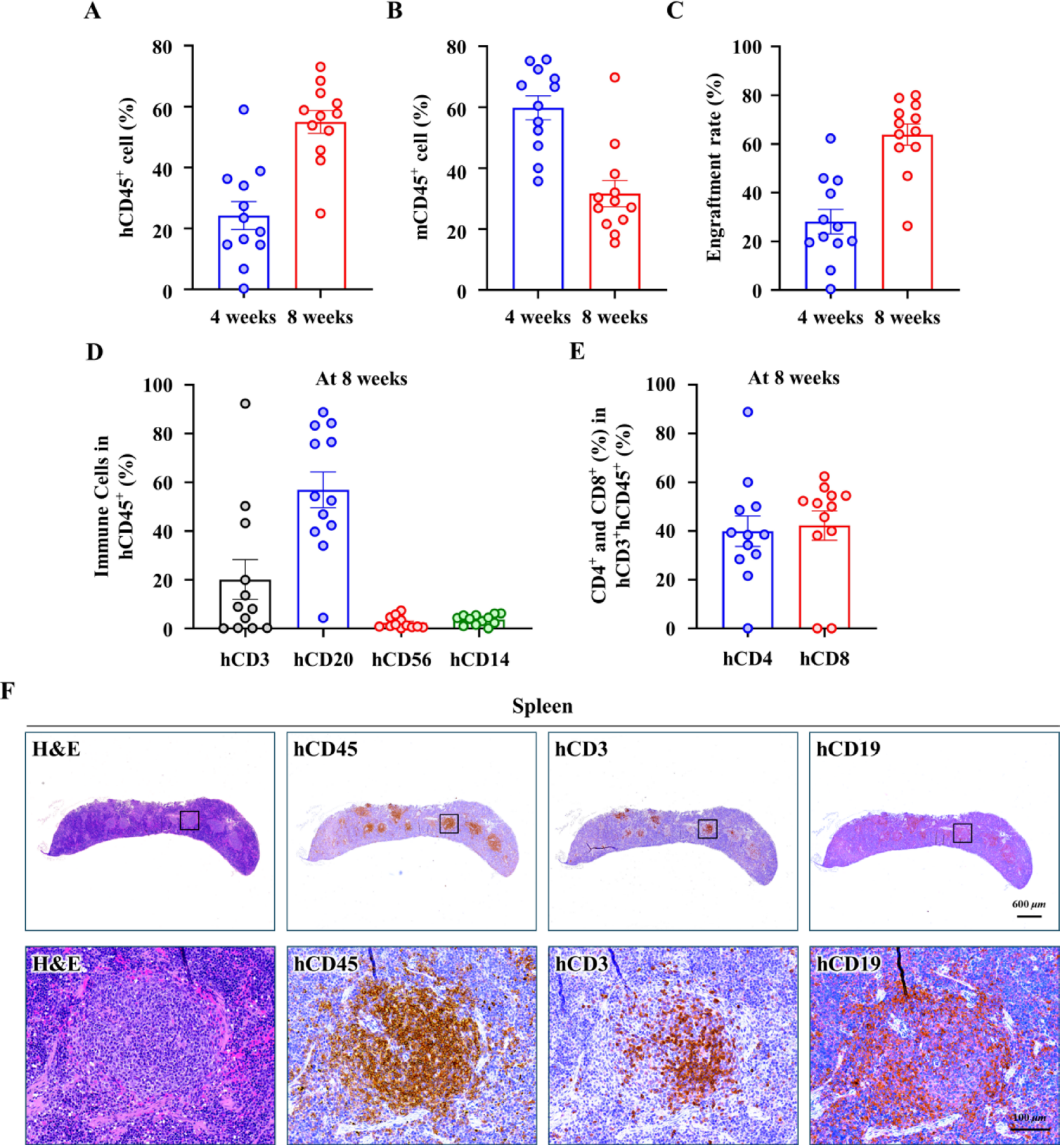
Validation and characterization of the HSC-humanized mouse model. (**A**) Proportion of hCD45^+^ cells in the blood at the indicated time points. (**B**) Proportion of mCD45^+^ cells in blood at the indicated time points. (**C**) Engraftment efficiency (% Graft) at different time points was calculated as the proportion of human CD45^+^ cells in the total CD45^+^ cell population (human and mouse) in peripheral blood. (**D**) Multilineage development of the human immune system in HSC-humanized mice. Flow cytometry analysis confirming the presence of human leukocytes (hCD45^+^), T cells (hCD3^+^), B cells (hCD20^+^), monocytes (hCD14^+^), and natural killer cells (hCD56^+^) within the hCD45^+^ cell population in peripheral blood. (**E**) Proportions of human CD4^+^ and CD8^+^ T cell subsets in hCD3^+^ cell populations. (**F**) Hematoxylin and eosin (H&E) and immunohistochemical staining for human leukocyte (hCD45^+^), T cell (hCD3^+^), and B cell (hCD19^+^) markers in the spleen of humanized mice. The upper panel displays low-magnification images (20 ×), whereas the lower panel shows high-magnification images (200 ×). Scale bars: low magnification, 600 µm; high magnification, 100 µm. Data are expressed as mean ± standard deviation. HSCs, hematopoietic stem cells.

The effectiveness of engraftment was assessed in every mouse to verify the humanization process. The humanized NOG mouse model was used to investigate PF. Four-day-old irradiated NOG pups were reconstituted with human CD34^+^ cord blood stem cells as described previously^28^. Multilineage development among human leukocytes in the blood was observed using flow cytometry analysis. The percentage of humanized mice successfully reconstituted with engrafted human immune systems was 63.86% ± 15.17% at 8 weeks after CD34^+^ cell injection (Fig. 3A–C), consistent with previously reported findings^35,36^. At 8 weeks after the humanization process, hCD3^+^, hCD4^+^, hCD8^+^, hCD20^+^, hCD14^+^, and hCD56^+^ cells were interspersed among hCD45^+^ cells (Fig. 3D,E), indicating the establishment of multiple human immune cell lineages. Following the development of a PF model, multilineage growth of human leukocytes, including T cells and B cells, in the secondary lymphoid organ (spleen) was observed in the same mouse (Fig. 3F).

These findings confirm that NOG mice reconstituted with human CD34^+^ HSCs exhibited robust engraftment and multilineage differentiation of human immune cells.

### Development of a PF model in HSC-humanized mice

**Fig. 4:**
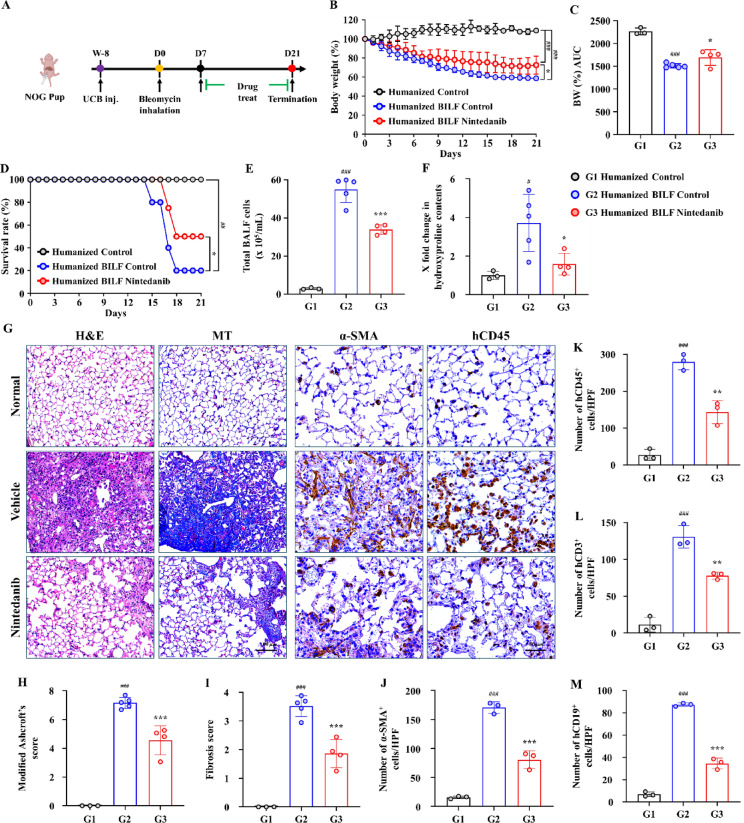
Establishment of BILF model in HSC-humanized mice and attenuation by nintedanib treatment of bleomycin-induced fibrosis. (**A**) An experimental design depicting the timeline for the humanization process, bleomycin administration, and subsequent nintedanib treatment. (**B**) Body weight percentage changes over time relative to day 0 in normal, vehicle-, and nintedanib-treated mice. (**C**) Area under the curve (AUC) of body weight percentage (BW %). (**D**) Survival curve comparing the vehicle and nintedanib treatment groups after bleomycin administration. (**E**) Data on total cell counts in the BALF. (**F**) Quantitative expression level (fold change compared with the normal group) of the hydroxyproline content in BILF lung tissue. (**G**) Representative hematoxylin and eosin (H&E) and Masson’s trichome (MT) staining of lung tissues, along with immunohistochemical (IHC) staining for α-SMA^+^ and hCD45^+^; H&E and MT, 200 × magnification; IHC, 400 × magnification. Scale bars: H&T and MT, 100 µm; IHC, 50 µm. (**H**) The average-modified Ashcroft’s score and (**I**) Fibrosis score, quantified from H&E and MT staining, respectively, indicating the extent of fibrosis. (**J**) Quantification data of α-SMA^+^ cells/high-power field (HPF). (**K**–**M**) Quantification of human immune cells, hCD45^+^ leukocytes, hCD3^+^ T, and hCD19^+^ T cells, indicated as the number of cells per HPF. Data are expressed as mean ± standard deviation. *P < 0.05, **P < 0.01, and ***P < 0.001 versus vehicle; ^#^ versus the normal group. BILF, bleomycin-induced lung fibrosis; PBMCs, peripheral blood mononuclear cells; BALF, bronchoalveolar lavage fluid; HSCs, hematopoietic stem cells.

To mimic human PF, a fibrosis model was established by administering BLM to humanized mice engrafted with human HSCs. The overall experimental design, including BLM administration and nintedanib treatment in HSC-humanized mice, is depicted in (Fig. 4A). BLM administration led to a steady decrease in body weight percentage, with BLM-treated mice exhibiting significantly greater weight loss than control mice (Fig. 4B). Quantitative analysis of body weight loss, represented by area under the curve (AUC), is presented in (Fig. 4C), demonstrating a significant reduction in BLM treated group. Survival analysis demonstrated a significant decrease in survival in the BLM-treated group compared with controls (Fig. 4D). BALF analysis revealed a pronounced increase in the total cell counts in the BLM-treated group (54.95 ± 6.81) compared with that in the control group (2.92 ± 0.47) (Fig. 4E). Moreover, BLM treatment resulted in a (3.71 ± 0.66)-fold increase in the hydroxyproline level in the lung tissue compared with that in the control group, indicating increased collagen production (Fig. 4F).

Histological analysis of lung tissues revealed significantly elevated Ashcroft’s scores (7.16 ± 0.38) and fibrosis scores (3.51 ± 0.36) in BLM-treated mice, consistent with the presence of severe PF (Fig. 4G–I). Immunohistochemical analysis confirmed the extensive infiltration of α-SMA^+^ cells (170.58 ± 9.87), highlighting ongoing fibrogenesis and myofibroblast activation (Fig. 4J). Furthermore, IHC for human-specific markers (hCD45, hCD3, and hCD19) confirmed the presence of human immune cell infiltration within lung tissues (Fig. 4K–M; Supplementary Fig. 3A–B).

**Fig. 5:**
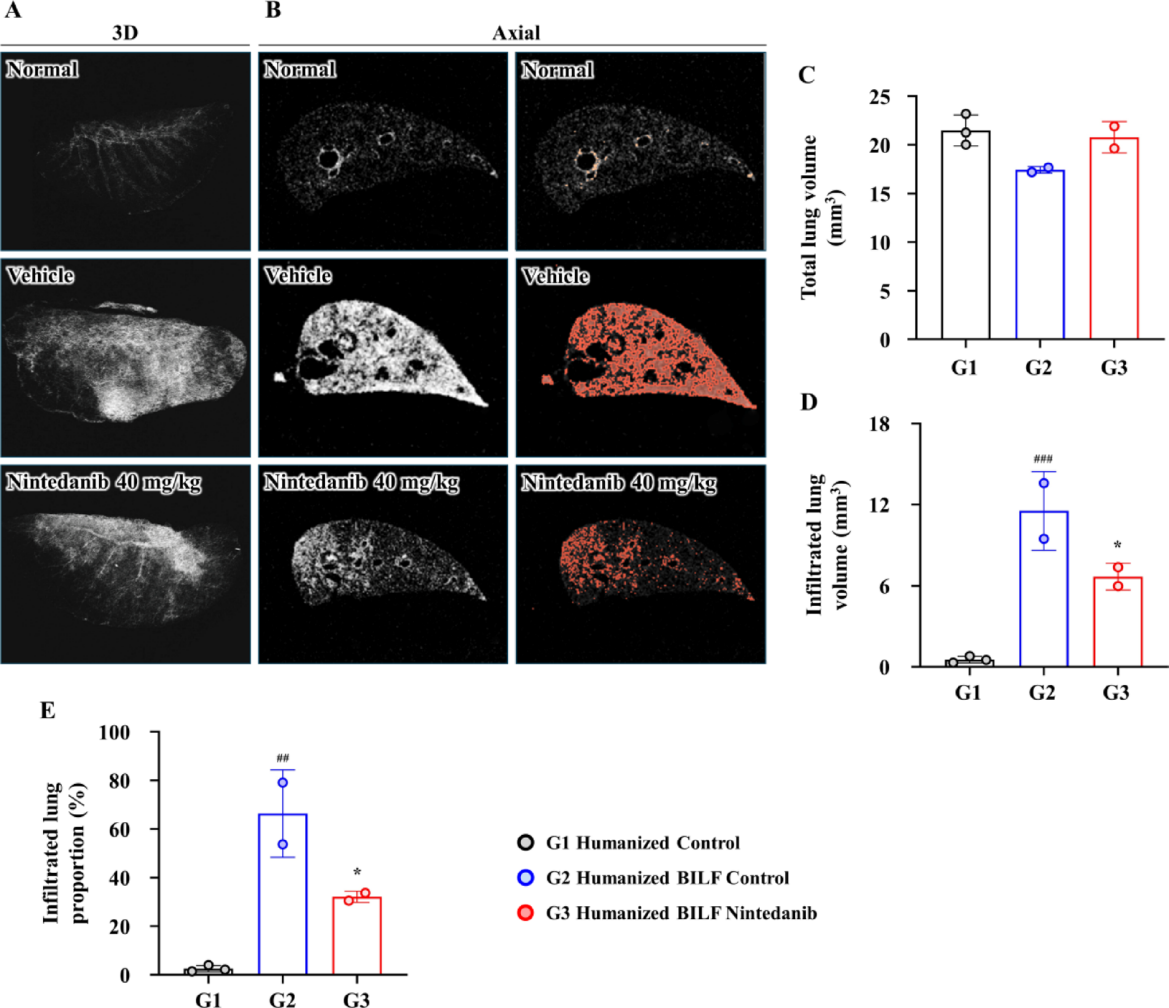
Ex vivo lung imaging was performed using micro-computed tomography (micro-CT) following bleomycin and nintedanib treatment in the HSC-humanized BILF model. (**A**) Representative 3D and (**B**) Axial views of micro-CT images of the left lung from the normal, vehicle-, and nintedanib-treated groups after bleomycin administration. (**C**) Total lung volume (mm^3^) measured from the micro-CT images. (**D**) Quantification of infiltrated lung volume, representing fibrosis volume (mm^3^). (**E**) Proportion of infiltrated lung calculated as the ratio of infiltrated lung volume to total lung volume. Data are expressed as mean ± standard deviation. *P < 0.05, **P < 0.01, and ***P < 0.001 versus vehicle; ^#^versus the normal group.

Furthermore, micro-CT analysis revealed extensive infiltration of the lungs with fibrotic changes (Fig. 5A–E).

Interestingly, the Normal (humanized control) group exhibited no signs of GvHD, as reflected by a Modified Ashcroft’s score and fibrosis score of 0 (Fig. 4H,I). This group also showed markedly lower infiltration of human immune cells, with hCD45^+^ cells (27.13 ± 14.83 per HPF) and hCD3^+^ cells (11.38 ± 10.07 per HPF) (Fig. 4K,L). Consistent with these findings, micro-CT analysis confirmed only minimal fibrotic involvement in the lungs (2.56 ± 1.31%) (Fig. 5E).

Overall, these results demonstrate that BLM induced PF in the HSC-humanized mouse model and closely replicated the pathophysiological development of lung fibrosis observed in human cases, while humanized control mice remained largely free of fibrosis and GvHD.

### Human immune cell infiltration in the lung tissues and BALF

Several experiments were conducted to investigate the involvement of human immune cells in the pathogenesis of fibrosis in humanized mice. In PBMC-humanized BILF mice, infiltration of human cells was confirmed via IHC, which revealed the presence of hCD45^+^ leukocytes and hCD3^+^ T cells in the lung tissues (Fig. 2E,F).

FACS analysis of the HSC-humanized mouse model revealed a pronounced immune response following BLM administration, with significant infiltration of human CD45^+^ leukocytes, human CD3^+^ T cells, and human CD20^+^ B cells in the BALF (Supplementary Fig. 3A–D). These findings were further validated through IHC staining of the lung tissues, which revealed the presence of human-specific markers, including hCD45^+^ leukocytes, hCD3^+^ T cells, and hCD19^+^ B cells. IHC staining further confirmed the increased presence of human immune cells in the HSC-humanized BILF models (Fig. 4K–M, Supplementary Fig. 2A–B). These findings are consistent with those of previous studies reporting the presence of T cells along with other cell types in the lungs and BALF of patients with PF^37–39^. These studies confirmed a significant immune response within the pulmonary environment, highlighting the role of T cells in the pathology of PF.

Our findings confirmed the presence of human cells in the lung and BALF, which are the main areas for the pathogenesis of PF. Therefore, human cells may contribute significantly to the development of PF in humanized mouse models.

### Nintedanib ameliorates BLM-induced PF in humanized mice

To validate our humanized BILF model, we subsequently assessed the therapeutic efficacy of the antifibrotic agent nintedanib in mitigating BLM-induced PF. Nintedanib treatment was initiated on day 7 after BLM administration in both PBMC- and HSC-humanized mouse models. Throughout the 14-day observation period, nintedanib effectively alleviated weight loss and improved survival outcomes in BLM-treated humanized mice (Figs. 1G,I and 4B,D). Furthermore, the total cell counts in the BALF significantly decreased in the nintedanib-treated group compared with those in the vehicle-treated group (Figs. 1J and 4E).

Histopathological examination showed that treatment with nintedanib significantly alleviated the severity of PF, as demonstrated by markedly lower modified Ashcroft’s and fibrosis scores in lung tissue than in the vehicle-treated control group (Figs. 2A–C and 4G–I). Immunohistochemical staining further demonstrated a significant reduction in human inflammatory cells (leukocytes, T cells, and B cells) in the lung tissue of the nintedanib-treated group (Figs. 2E,F and 4K–M).

These results indicated that nintedanib, significantly alleviated the fibrotic and inflammatory burden associated with BLM-induced PF in humanized mouse models, highlighting its potential therapeutic efficacy and validating our humanized BILF model. Our findings are consistent with those observed in the conventional BILF model (Supplementary Fig. 4A–L), where nintedanib effectively mitigated fibrosis.

## Discussion

This research emphasizes the need to establish humanized BILF models to circumvent the species-specific constraints of conventional animal models, particularly in the assessment of human-specific monoclonal antibody therapeutics and cell therapies. To achieve this, we herein successfully established and validated PBMC- and HSC-humanized mouse models for PF. The PBMC-humanized BILF model effectively replicated the key pathological features of human PF, including immune cell infiltration, fibroblast activation, and extracellular matrix deposition. However, its utility was limited by the onset of GvHD and spontaneous lung fibrosis, as observed in the normal humanized control mouse group. In contrast, the HSC-humanized BILF model demonstrated superior multilineage human immune cell engraftment, remained free of GvHD until the end of the study (11 weeks), and exhibited a robust fibrotic response, demonstrating a more reliable platform for translational studies. The presence of human immune cells within the lung tissue and BALF underscores their active role in PF progression, which aligns with clinical observations^37–39^. However, the lung parenchyma in our model remains murine, with epithelial cells, fibroblasts, and endothelial cells of mouse origin. Since these cell types play central roles in extracellular matrix deposition, epithelial–mesenchymal transition, and fibroblast activation, this limits full replication of human PF pathophysiology. Future refinements could include co-engraftment of human lung tissue xenografts, transplantation of patient-derived airway or alveolar organoids, or genetic replacement of key murine pathways with human counterparts to further humanize the lung microenvironment. Despite these limitations, the HSC-humanized BILF model has immediate value for preclinical testing of human-specific immune-modulating therapies, such as monoclonal antibodies, cytokine inhibitors, and adoptive cell therapies, which cannot be adequately evaluated in murine-only models due to species-specific immune differences. By bridging the gap between in vitro assays and clinical trials, this model can accelerate the translational pipeline for immune-targeted PF therapies.

Researchers have increasingly advocated for the prioritization of human immunology over traditional mouse models to enhance the clinical translatability of immunological research findings^40,41^. Monoclonal antibodies and cell therapies are designed to target human antigens or interact with human immune cells. Differences in antigen structures and immune cell signaling between humans and mice can lead to the failure of these therapies in inducing the desired efficacy or mechanisms of action in traditional murine models^10–13^.

These research gaps highlight the limitations of exclusively using traditional murine models and underscore the need of humanized models that more accurately mimic human immune responses and diseases^18^.

Several models of humanized PF, often using human lung fibroblasts or PBMCs collected from patients, leverage the immunodeficient nature of host mice to enable human cell engraftment^19,20,42–44^. These models have enhanced the understanding of IPF pathogenesis and facilitated investigations of human-specific antifibrotic therapies. However, fibroblast-based models lack immune cells, limiting their ability to replicate the immune–fibroblast interactions critical to IPF^7^. A previous study established a BILF model using PBMC-based humanized mice to study PF^13^. While valuable, this model has notable limitations. According to other previous studies, GvHD onset typically occurs approximately 5 weeks after PBMC injection, coinciding with fibrosis development, which occurs at approximately 8 weeks post injection^21,22^. The overlapping emergence of GvHD and fibrosis introduces confounding variables that complicate the analysis of fibrosis-specific processes, creating difficulty in distinguishing between the contributions of immune-driven fibrosis and systemic immune pathology.

To address this gap, the present study established a robust experimental BILF model using humanized mice to advance the understanding of IPF and evaluate proposed therapies. Using a stepwise approach, we established fibrosis models using PBMC- and HSC-humanized mouse models. To overcome the challenges of GvHD in the PBMC-humanized model, we optimized the experimental timeline by restricting the study to 5 weeks after PBMC injection based on the onset time of GvHD determined in a previous study^21^. BLM was administered 2 weeks after PBMC reconstitution, and samples were collected 3 weeks later. This approach ensured an adequate study window for fibrosis development while reducing the potential impact of GvHD on the experimental results. To induce PF in humanized mice, we adopted the optimized OA method in conjunction with BLM, based on its robust and consistent fibrotic response^7,45^.

The results of the PBMC-humanized BILF model demonstrated that BLM induced significant PF, which was characterized by a notable body weight loss, decreased survival rate, severe lung damage, and substantial collagen deposition. The presence of human CD45^+^ leukocytes and CD3^+^ T cells in the lung tissues indicated active human immune cell infiltration, emphasizing their role in fibrosis progression. Although reducing the study window to 5 weeks reduced the risk of GvHD, the normal humanized control group still developed GvHD and baseline fibrosis, as confirmed by histology, immunohistochemistry, and micro-CT analysis. The model’s reliance on T cell engraftment further limited its ability to capture the full spectrum of human immune responses.

To overcome these obstacles, we developed a BILF model in HSC-humanized mice, enabling multilineage human hematopoietic cell engraftment, including T cells, B cells, natural killer cells, and monocytes^18^. Accordingly, we performed the intrahepatic injection of CD34^+^ HSCs into 4-day-old irradiated pups, since newborn NOD mice provide optimal conditions for supporting human hematopoietic cell engraftment^46^. This model provides a comprehensive representation of human immune components while significantly reducing the risk of GvHD, thereby offering a stable platform for studying PF and evaluating therapies. BLM administration successfully induced PF in HSC-humanized mice. The model was validated based on significant changes in body weight percentage and increased mortality as well as using various analyses, including BALF cell counts, histological assessment, hydroxyproline assay, and micro-CT imaging, which collectively confirmed severe fibrosis with consistent and reproducible fibrotic responses. Human immune cell infiltration was evident in the BALF and lung tissues, as indicated by the presence of T cells (both CD4^+^ and CD8^+^ T cells) and B cells, emphasizing their critical role in fibrosis progression. These findings were consistent with those of previous clinical studies that highlighted the importance of BALF analysis in understanding the pathogenetic mechanisms underlying UIP/IPF^37^. The detection of CD8^+^ T lymphocytes in the BALF has been associated with certain physiological and clinical parameters, emphasizing their role in IPF pathogenesis. Furthermore, IHC studies have revealed a strong correlation between CD8^+^ T lymphocytes and functional and clinical markers of disease severity, indicating their potential as biomarkers for disease progression^38^. T cells as well as other cell types occur in the lungs and BALF of individuals with PF^39^.

To validate our humanized BILF model, we treated both models with nintedanib, as a positive control drug. Nintedanib was chosen as the reference antifibrotic agent due to its well-characterized, broad-spectrum tyrosine kinase inhibition of PDGFRs, FGFRs, and VEGFRs—critical pathways in fibroblast activation and extracellular matrix deposition in pulmonary fibrosis^47,48^. Beyond its antifibrotic effects, Nintedanib has demonstrated anti-inflammatory properties by reducing immune cell infiltration and suppressing pro-inflammatory cytokines in preclinical lung injury models^49^. Although Pirfenidone also possesses antifibrotic and anti-inflammatory activities, primarily via modulation of TGF-β signaling and cytokine production^24^, Nintedanib was prioritized in this study for its multi-target mechanism, reproducible efficacy across diverse fibrosis models, and well-defined pharmacodynamics, which facilitate translational relevance to human IPF therapy. Nintedanib treatment effectively ameliorated fibrosis in both models, inhibited body weight loss, and improved the survival rate. Nintedanib significantly reduced the total BALF cell counts, modified Ashcroft’s score, fibrosis scores, α-SMA^+^ cell counts, and collagen levels, indicating a substantial reduction in the fibrotic burden. Furthermore, nintedanib decreased human immune cell infiltration in lung tissues, as evidenced through IHC analyses. FACS analysis of the BALF revealed a decrease in human CD45^+^ leukocytes and CD3^+^ T cells but an increase in CD20^+^ B cells. Within the CD3^+^ T cell population, the number of CD8^+^ cytotoxic T cells decreased, whereas the number of CD4^+^ helper T cells relatively increased. These findings demonstrate the efficacy of nintedanib as an anti-inflammatory agent, highlighting its immunomodulatory effects in mitigating the progression of immune-driven fibrosis in the humanized BILF model. Furthermore, these results are consistent with those of a previous study reporting reduced lymphocyte accumulation in the BALF, reinforcing the therapeutic potential of nintedanib in fibrosis management^50^.

By demonstrating anti-inflammatory, antifibrotic, and immunomodulatory effects, our results provide further evidence supporting the efficacy of nintedanib in alleviating fibrosis progression in the humanized BILF model. Our findings are consistent with those observed in the conventional BILF model, where nintedanib effectively mitigates fibrosis. The significant reduction in fibrosis observed in our study following nintedanib treatment underscores the successful establishment of the BILF model in humanized mice.

## Conclusion

Overall, this study emphasizes the critical role of humanized BILF models in bridging the gap between preclinical research and clinical application. The HSC-humanized mouse model, established for the first time in this study, provides a robust platform for studying human lung fibrosis by closely mimicking the disease’s pathophysiology, including the recruitment of human leukocytes to the lung tissue and BALF, within a multilineage human immune cell environment and the absence of GvHD onset. The study further demonstrates that antifibrotic treatment significantly reduces both the fibrotic burden and human leukocyte infiltration, highlighting the model’s potential for evaluating targeted therapies.

In the future, this humanized model will provide an invaluable platform for testing the efficacy of human-specific therapeutics, such as monoclonal antibodies and cell therapies.

## Supplementary Information

Below is the link to the electronic supplementary material.

Supplementary Material 1

## Data Availability

All data generated or analyzed during this study are included in this published article and its accompanying supplementary information files.
