# Supplementary material for: Establishment of a humanized mouse model of pulmonary fibrosis for advancing drug validation strategies
Source: Sci Rep. 2025 Oct 6;15:34748. doi: 10.1038/s41598-025-18370-7 (PMC12501095; doi:10.1038/s41598-025-18370-7)
Supplement: Supplementary file 1 — Supplementary Material 1 [file 41598_2025_18370_MOESM1_ESM.docx]

**Supplementary information**

**Supplementary Table 1: List of antibodies used for FACS analysis.**

| **Antibody** | **Conjugation** | **Company** |
| --- | --- | --- |
| Anti-mouse CD45 | PerCP | Biolegend |
| Anti-human CD45 | APC |  |
| Anti-human CD3 | FITC |  |
| Anti-human CD4 | PerCP |  |
| Anti-human CD8 | PE |  |
| Anti-human CD20 | PE-Cy7 |  |
| Anti-human CD14 | PE-Cy7 |  |
| Anti-human CD56 | PE |  |

**Supplementary Table 2: Modified Ashcroft’s score criteria.**

| **Alveolar septa** | | **Score** | | **Lung structure** |
| --- | --- | --- | --- | --- |
| No fibrotic burden at the most flimsy small fibers in some alveolar walls | 0 | | Normal lung | |
| Isolated gentle fibrotic changes (septum ≤3× thicker than normal | 1 | | Alveoli partly enlarged and rarefied, but no fibrotic masses present | |
| Clearly fibrotic changes (septum >3× thicker than normal) with knot-like formation but not connected to each other | 2 | | Alveoli partly enlarged and rarefied, but no fibrotic masses | |
| Contiguous fibrotic walls (septum >3× thicker than normal) predominantly in whole microscopic field | 3 | | Alveoli partly enlarged and rarefied, but no fibrotic masses | |
| Variable (>50% septa exist) | 4 | | Single fibrotic masses (≤10% of microscopic field) | |
| Variable (20%-50% septa exist) | 5 | | Confluent fibrotic masses (>10% and ≤50% of microscopic field). Lung structure severely damaged but still preserved | |
| Variable (<20% septa exist), mostly not existent | 6 | | Large contiguous fibrotic masses (>50% of microscopic field). Lung architecture mostly not preserved | |
| Non-existent | | 7 | | Alveoli nearly obliterated with fibrous masses but still up to five air bubbles |
| Non-existent | | 8 | | Microscopic field with complete obliteration with fibrotic masses |

**Supplementary Table 3: Fibrosis score criteria.**

| **Score** | **Description** |
| --- | --- |
| 1 | < 25% area are fibrosis among the whole field |
| 2 | 25–50% area is fibrosis among the whole field |
| 3 | 50–75% area is fibrosis among the whole field |
| 4 | 75–100% area is fibrosis among the whole field |

**Supplementary Table 4: List of antibodies used for IHC staining.**

| **Antibody** | **Company** |
| --- | --- |
| Rat Anti Human CD45 Monoclonal Antibody  (Human Specific) | SYSY-HistoSure |
| Rat Anti Human CD3 Monoclonal Antibody  (Human Specific) |  |
| Rat Anti Human CD19 Monoclonal Antibody  (Human Specific) |  |


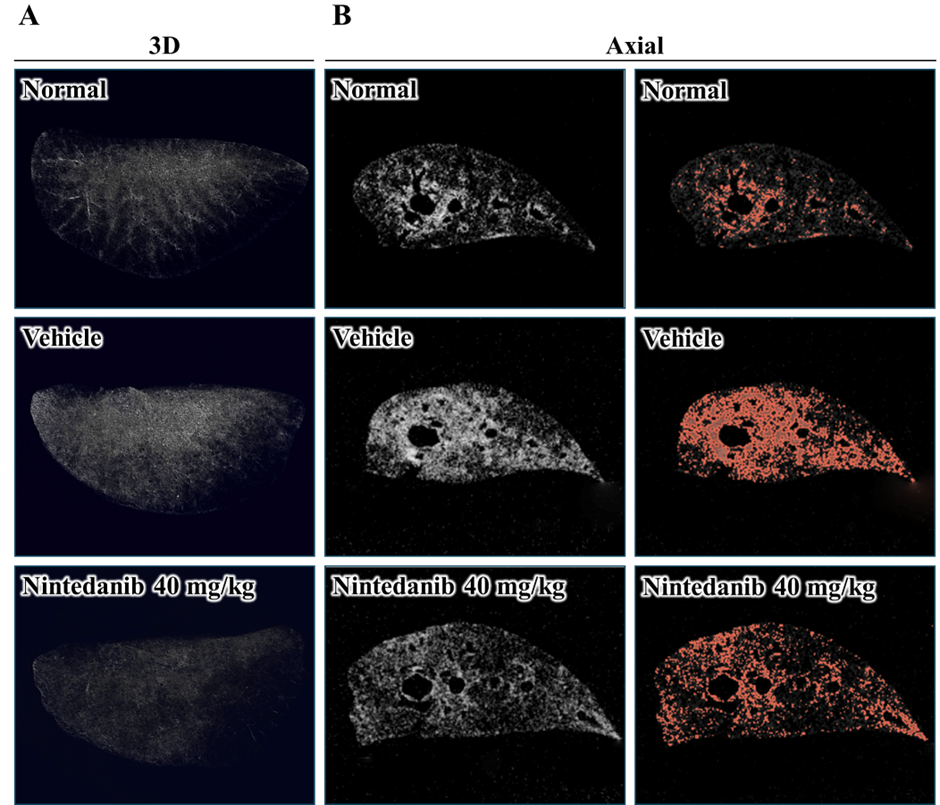


**Supplementary Fig. 1 *Ex vivo* lung assessment using micro-CT following bleomycin administration and nintedanib treatment in the PBMC-humanized BILF model.** Representative **(A)** 3D and **(B)** Axial views of micro-CT images showing the left lung from the normal, vehicle, and nintedanib-treated groups after bleomycin administration.


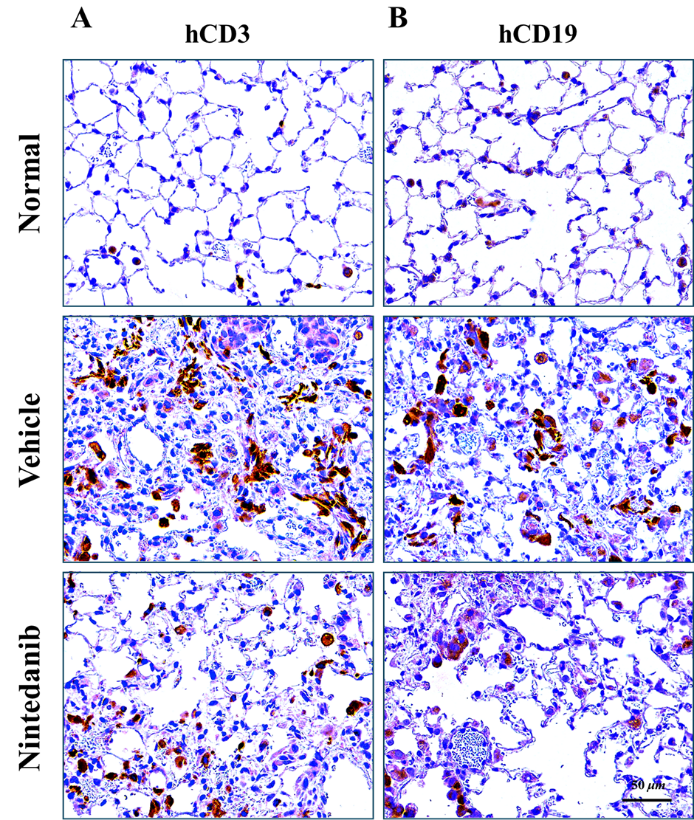


**Supplementary Fig. 2 Human immune cells infiltrated in the lung tissue of HSC-humanized BILF mice. (A)** Representative images of lung sections stained for human CD3 (hCD3^+^) to identify human T cells. **(B)** Representative images stained for human CD19 (hCD19^+^) to identify B cells. Scale bars: 50 µm for x400 magnification.


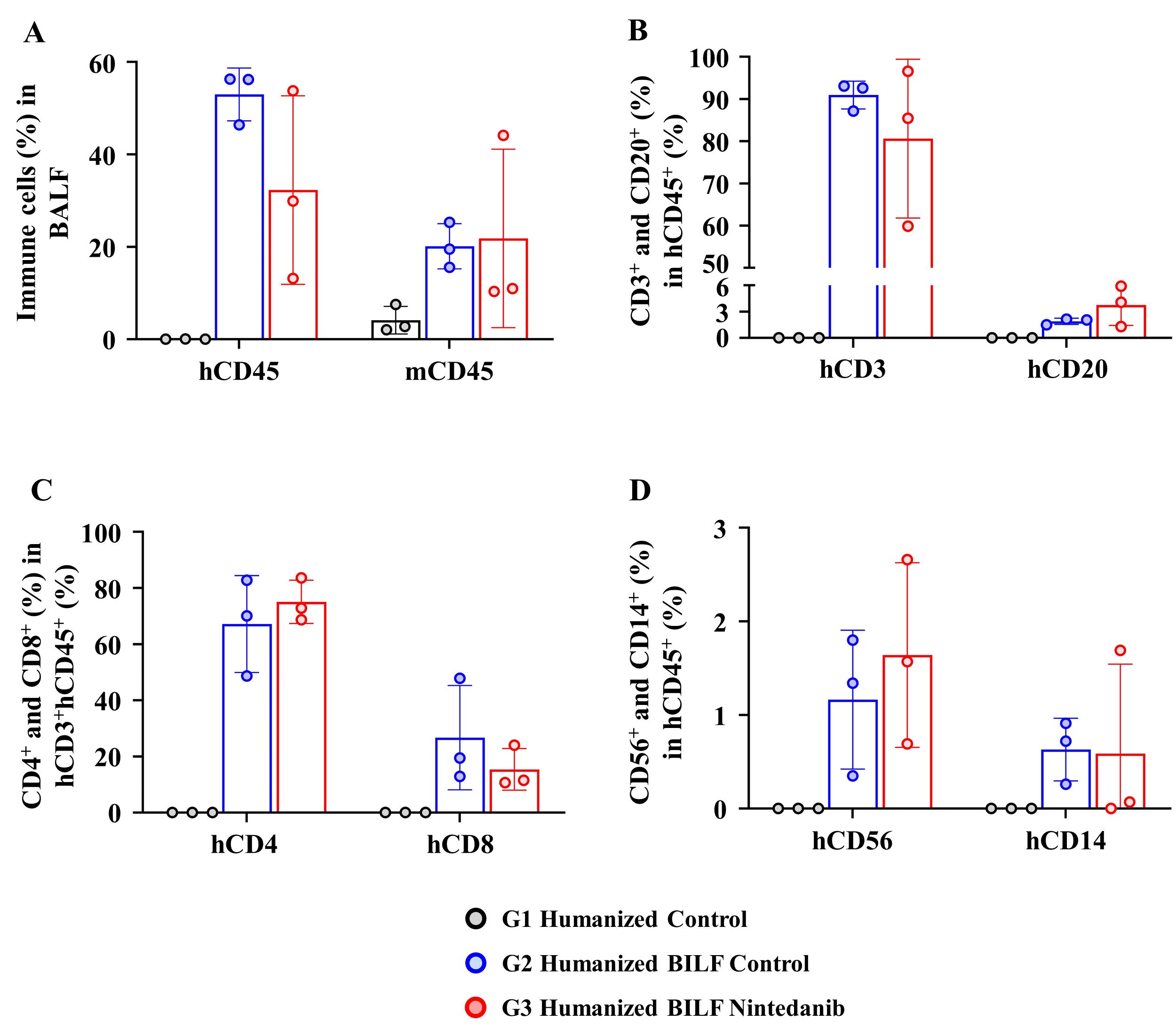


**Supplementary Fig. 3 FACS analysis of BALF of HSC-humanized mouse after BILF model. (A)** Proportion of human and mouse CD45^+^ cells in BAL fluid. **(B)** Proportion of human hCD3^+^ T and hCD20^+^ B cells in hCD45^+^ cells. **(C)** Proportion of human CD4^+^ T and CD8^+^ T cells in hCD45^+^hCD3^+^cells. **(D)** Proportion of human hCD56^+^ NK and hCD14^+^ monocyte cells in hCD45^+^ cells. The mice were grouped into Normal and BILF mice treated with Vehicle, and Nintedanib (40 mg/kg, QD, PO). Data are expressed as the mean ± SD.


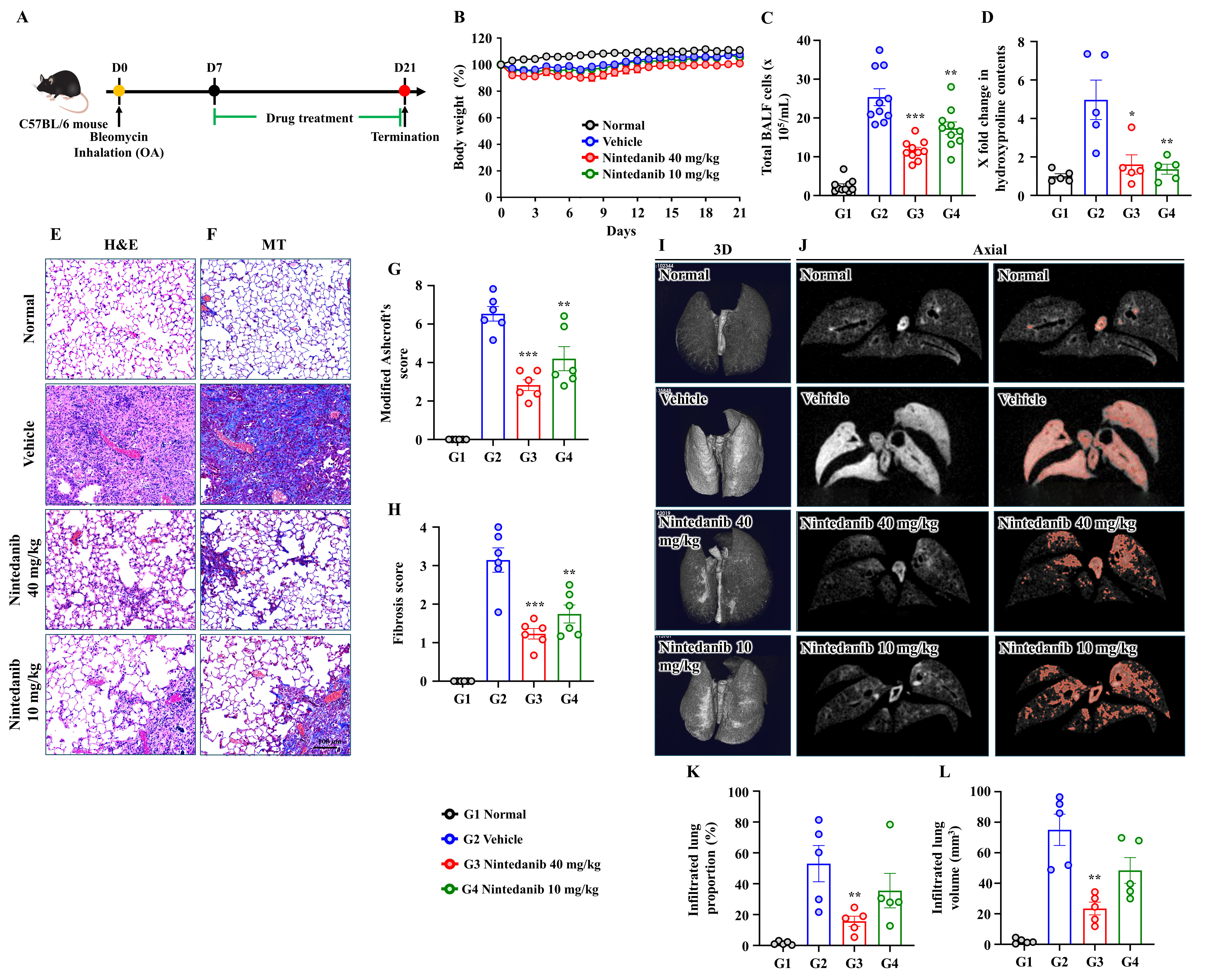


**Supplementary Fig. 4 Bleomycin administration successfully induced pulmonary fibrosis in C57BL/6 mice, while treatment with nintedanib significantly mitigated bleomycin-induced fibrosis. (A)** Schematic overview of experimental procedures for the C57BL/6 BILF mouse model. **(B)** Percentage variation in body weight from day 0 to day 21, relative to initial values. **(C)** Total BALF cells count. **(D)** Hydroxyproline content in lung tissues from each group was measured to assess collagen deposition. **(E)** Representative images of H&E and **(F)** MT at x200 magnification. 100 µm. **(G)** Average modified Ashcroft’s score and **(H)** Fibrosis score derived from H&E and MT stain respectively. **(I)** Representative 3D and **(J)** Axial view of *ex-vivo* lung micro-CT imaging. **(K)** Infiltrated lung proportion (%) calculated based on total lung volume and fibrosis volume and infiltrated lung volume (mm^3^) in each group. Data are expressed as the mean ± SEM. * = P < 0.05, ** = P < 0.01, and *** = P < 0.001 versus Vehicle.

**List of abbreviations**

AUC Area under the curve

BALF Blood and bronchoalveolar lavage fluid

CT Computed tomography

FACS Fluorescence-activated cell sorter

GvHD Graft-versus-host disease

H&E Hematoxylin and eosin

HPF High-power field

HSC Hematopoietic stem cell

IPF Idiopathic pulmonary fibrosis

MT Masson’s trichrome

OA Oropharyngeal aspiration

PBMC Peripheral blood mononuclear cells

RPMI Roswell Park Memorial Institute

UCB Umbilical cord blood

UIP Usual interstitial pneumonia
